# Macrocolony of NDM-1 Producing *Enterobacter* *hormaechei* subsp. *oharae* Generates Subpopulations with Different Features Regarding the Response of Antimicrobial Agents and Biofilm Formation

**DOI:** 10.3390/pathogens8020049

**Published:** 2019-04-14

**Authors:** Flávia Roberta Brust, Luana Boff, Danielle da Silva Trentin, Franciele Pedrotti Rozales, Afonso Luís Barth, Alexandre José Macedo

**Affiliations:** 1 Faculty of Pharmacy and Center of Biotechnology, Federal University of Rio Grande do Sul, Porto Alegre, Rio Grande do Sul CE 90610-000, Brazil; flaviabrust@gmail.com (F.R.B.); luana_boff@hotmail.com (L.B.); 2 Basic Health Sciences Department, Federal University of Health Sciences of Porto Alegre, Porto Alegre, Rio Grande do Sul CE 90050-170, Brazil; danistrentin@gmail.com; 3 Laboratory of Research in Bacterial Resistance, Center for Experimental Research, Clinical Hospital of Porto Alegre, Federal University of Rio Grande do Sul, Porto Alegre, Rio Grande do Sul CE 90035-007, Brazil; frozales@hotmail.com (F.P.R.); albarth@hcpa.edu.br (A.L.B.)

**Keywords:** *Enterobacter*, macrocolony biofilm, *bla*_NDM-1_, antimicrobial treatment, *Galleria mellonella*

## Abstract

*Enterobacter cloacae* complex has been increasingly recognized as a nosocomial pathogen representing the third major Enterobacteriaceae species involved with infections. This study aims to evaluate virulence and antimicrobial susceptibility of subpopulations generated from macrocolonies of NDM-1 producing *Enterobacter hormaechei* clinical isolates. Biofilm was quantified using crystal violet method and fimbrial genes were investigated by PCR. Susceptibility of antimicrobials, alone and combined, was determined by minimum inhibitory concentration and checkerboard assays, respectively. Virulence and efficacy of antimicrobials were evaluated in *Galleria mellonella* larvae. Importantly, we verified that some subpopulations that originate from the same macrocolony present different biofilm production ability and distinct susceptibility to meropenem due to the loss of *bla*_NDM-1_ encoding plasmid. A more in-depth study was performed with the 798 macrocolony subpopulations. Type 3 fimbriae were straightly related with biofilm production; however, virulence in larvae was not statistically different among subpopulations. Triple combination with meropenem–rifampicin–polymyxin B showed in vitro synergistic effect against all subpopulations; while in vivo this treatment showed different efficacy rates for 798-1S and 798-4S subpopulations. The ability of multidrug resistant *E. hormaechei* isolates in generating bacterial subpopulations presenting different susceptible and virulence mechanisms are worrisome and may explain why these infections are hardly overcome.

## 1. Introduction

The Enterobacteriaceae family is composed by many bacteria, including *Escherichia coli*, *Klebsiella* spp., and *Enterobacter* spp., responsible for community-associated as well as healthcare-associated infections [1,2,3]. In the last decades, species of the *Enterobacter cloacae* complex (ECC) have aroused greater concern, since they are increasingly associated with carbapenemase-encoding genes acquisition, being the second or third most common carbapenemase-producing Enterobacteriaceae (CPE) [4,5,6,7]. Carbapenem resistance constitutes a global public-healthcare problem associated with a high mortality and an increase of healthcare costs [8,9,10]. The New Delhi metallo-β-lactamase-1 (NDM-1) is one of the most clinically significant carbapenemase, this enzyme has been rapidly spread through all continents [11] since its detection in 2008 in India [12]. 

Therapy options for CPE are extremely limited and the optimal treatment for these infections is controversial. Studies have reported that combination therapy with two or more antimicrobials is associated with a better outcome than monotherapy [13,14,15]. The synergistic interactions among antimicrobial agents minimize the use of extremely high doses and emergence of resistance, as well as potentiate the effectiveness of individual agents [16]. Frequently, carbapenem-containing combinations are used in addition to polymyxin in clinical settings. Studies suggest an additional benefit of including a carbapenem in combination regimens, especially in the context of strains with low minimum inhibitory concentrations (MICs) against carbapenems [15,17]. Rifampicin is also considered for inclusion in combination regimens because of its ability to penetrate intracellular sites and biofilms, which could be important in the treatment of CPE infections involving biofilm [16]. Susceptibility to fluoroquinolones is rare among CPE, while susceptibility to sulfas and aminoglycosides may be unpredictable and can vary according to geographic location and strain type. Antimicrobials with reliable activity against CPE (> 85% of strains susceptible) typically include tigecycline, polymyxin B and colistin, and fosfomycin [17].

Most studies involving CPE evaluate treatment options against *Klebsiella pneumoniae* carbapenemase (KPC) infections. Studies of antimicrobial agent combinations against NDM-1 strains remains small [17]. In order to evaluate the best therapeutic options for bacterial infections, the non-vertebrate *Galleria mellonella* has been increasingly used as experimental host since this model is inexpensive, there are no ethical constraints, and it represents an easy alternative to mammalians to generate reliable and reproducible data about bacterial pathogenicity and antimicrobial treatment [18,19].

The taxonomy of ECC is confusing, and uncertainty still remains about what species belong to this complex [4]. ECC is composed by closed related species and subspecies; one of the most frequently species isolated in humans is *Enterobacter hormaechei* [4,20,21,22,23]. *Enterobacter hormaechei* strains can persist and spread in nosocomial environments, and often exhibit resistance to multiple clinically important antimicrobial agents [24]. This species has been also related with hospital outbreaks [24,25]. Although *E. hormaechei* is an important emerging pathogen and a key member of the highly diverse ECC [26], little is known about their virulence-associated properties [23,27]. Paauw et al. (2009) evaluated an *E. hormaechei* strain that caused an outbreak in Netherlands and found most isolates carried large conjugative plasmid containing genes encoding heavy-metal resistance, mobile elements, pili-associated proteins and exported proteins, as well as multiple-resistance genes [25].

Biofilm formation represents an important virulence mechanism produced by bacteria. The microbial multicellular relationship produces physical structures reflecting the complex interactions among their individual constituents. Various bacterial activities, including cell growth and cell death, nutrient acquisition, waste product accumulation, motility mechanisms, and exopolysaccharide synthesis can influence the biofilm architecture and functionality [28,29]. Like in biofilms, bacteria grown on agar surfaces represent heterogeneous and spatially structured populations, where some cells are actively growing, whereas others are in stationary or intermediate stages [30]. These characteristics make bacterial macrocolony a highly valuable model to study physiological differentiation and architectural development in biofilm [29].

Considering the importance of ECC in healthcare-associated infections and its ability to acquire antimicrobial-resistance genes, the aim of this study was to evaluate virulence and response to antimicrobial agents of subpopulations generate by macrocolony biofilm of NDM-1 producing *E. hormaechei* subsp. *oharae* clinical isolates. 

## 2. Results

### 2.1. Bacterial Macrocolonies Generate Subpopulations with Distinct Susceptibility to Meropenem and Ability to Form Biofilm

**Figure 1:**
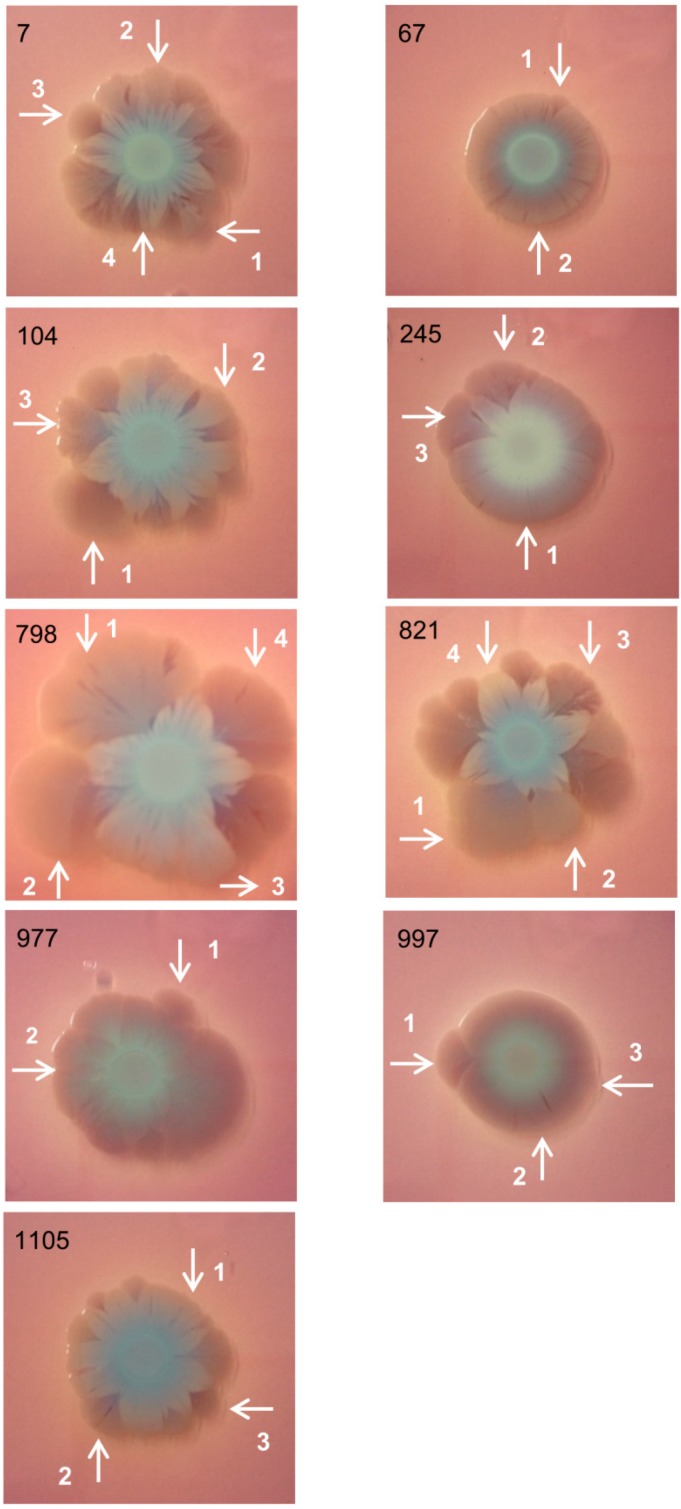
Macrocolonies of *E. hormaechei* subsp. *oharae* clinical isolates. Bacterial macrocolonies were grown in LB with Congo Red and Coomassie brilliant blue for 5 days at 37 °C. The white arrows and numbers indicated areas of macrocolonies, termed subpopulations, used for further experiments. These subpopulations were randomly selected according their morphologies.

After five days of incubation, *E. hormaechei* subsp. *oharae* macrocolonies generated distinguishable heterogeneous regions, termed subpopulations (Figure 1). These subpopulations were randomly selected according their morphologies and cells taken from them were used in the follow experiments. Subpopulations originated from the same macrocolony showed differences in meropenem susceptibility; some were susceptible (MIC < 2 µg/mL) and others resistant (MIC > 8 µg/mL). Presence of *bla*_NDM-1_ gene was evaluated in all subpopulations (data not shown) and it was not detected in susceptible subpopulations, confirming that the loss of this gene restored meropenem susceptibility. Susceptibility to ceftazidim, ciprofloxacin, gentamicin, and meropenem are summarized in Table 1. Subpopulations of all macrocolonies were resistant to ceftazidim (MIC > 4 ug/mL) and ciprofloxacin (MIC > 0.5 ug/mL) and only subpopulations originated of macrocolonies 7 and 997 were susceptible to gentamicin (MIC < 2 µg/mL), according the European Committee on Antimicrobial Susceptibility Testing (EUCAST) [31]. MIC values of the antimicrobial agents cited above were similar among subpopulations of the same macrocolony. 

**Figure 2:**
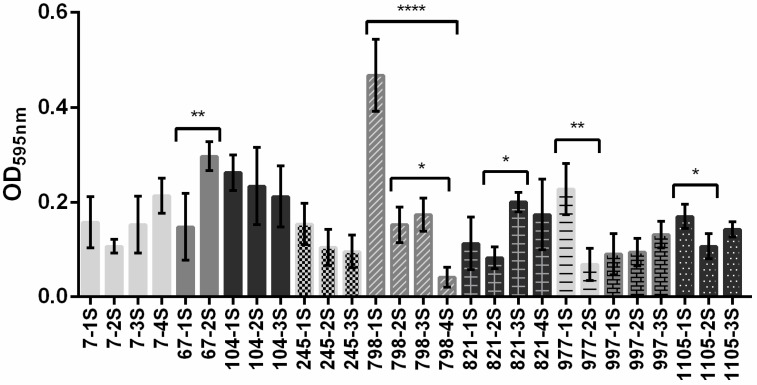
Biofilm formation of macrocolony subpopulations of *E. hormaechei* subsp. *oharae* clinical isolates. Bacteria were grown at 37 °C in microtiter plates containing Tryptone Soya Broth (TSB) for 24 h and then biofilm formation was quantified using crystal violet assay. The results are presented as the means and standard deviation. Significant differences in biofilm formation, among subpopulations of the same macrocolony, were pointed out when *p* < 0.05 (*), *p* < 0.01 (**), *p* < 0.0001 (****).

Results of crystal violet assays showed that subpopulations were not able to produce much biofilm; however, we could still observe statistically differences in the ability to form biofilm among subpopulations of five macrocolonies (67, 798, 821, 977, and 1105) (Figure 2). Considering that subpopulations of the group 798 showed a heterogeneous behavior in biofilm formation and 798-1S was the highest biofilm producer, this group (798-1S, 798-2S, 798-3S, and 798-4S) was selected for further experiments in this study.

### 2.2. Subpopulations of 798 Macrocolony

#### 2.2.1. Type 3 Fimbriae are Important for Biofilm Formation in *E. hormachei* subsp. *oharae*

**Figure 3:**
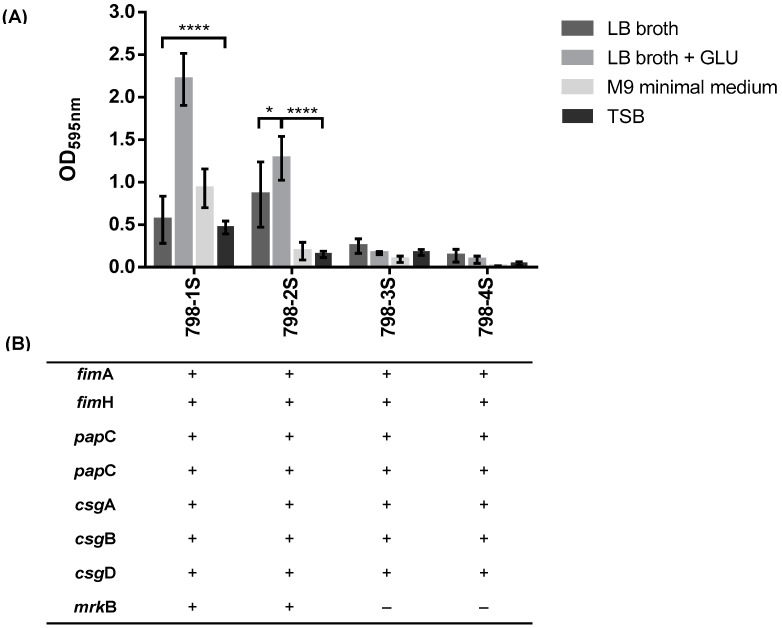
(**A**) Biofilm formation of *E. hormaechei* subsp. *oharae* 798 group. Bacteria were grown at 37 °C in microtiter plates containing LB broth, LB broth supplemented with glucose, M9 minimal medium or TSB for 24 h, after which biofilm formation was quantified. The results are presented as the means and standard deviation for three independent experiments. Significant differences in biofilm formation using LB broth + GLU were pointed out: *p* < 0.05 (*) and *p* < 0.0001 (****). (**B**) Fimbrial encoding genes: *fim*A, *fim*H (type 1 fimbriae genes), *pap*C, *pap*D (P pili genes), *csg*A, *csg*B, *csg*D (curli genes), and *mrk*B (type 3 fimbriae gene). Gene present: +; and gene absent: –.

Biofilm formation was determined in different media: Luria Bertani broth (LB), LB with glucose (0.02 M), and M9 minimal medium supplemented with glucose (0.02 M) and MgSO_4_ (0.002 M). The results showed that two (798-1S and 798-2S) of the four subpopulations were able to form biofilm (Figure 3A) and the amount of biofilm produced was higher in LB supplemented with glucose.

Since fimbriae play an important role in attachment and biofilm formation in gram-negative bacteria, we investigated the presence of fimbrial genes in order to understand the difference in biofilm formation ability among subpopulations. Genes encoding curli (*csg*A, *csg*B, and *csg*D), type 1 fimbriae (*fim*A and *fim*H), and P pili (*pap*C and *pap*D) were detected in all subpopulations. However, type 3 fimbriae encoding gene (*mrk*B) was detected only in two subpopulations (798-1S and 798-2S) (Figure 3B). Interestingly, only subpopulations with *mrk*B encoding gene were able to produce biofilm. 

#### 2.2.2. Checkerboard Assay: Triple Combination (meropenem–rifampicin–polymyxin B) is Effective against All Subpopulations

Synergism with double and triple antimicrobial combination against subpopulations generated from the 798 macrocolony was evaluated using checkerboard assay. At first, MIC of meropenem, rifampicin, and polymyxin B was determined. According to these results, all subpopulations showed a similar antimicrobial profile: susceptible to polymyxin B, resistant to meropenem, and with high MIC for rifampicin (128 µg/mL) (Table 2). In vitro combined inhibitory activities of meropenem–polymyxin B and rifampicin–meropenem achieved synergy against only one subpopulation (798-1S). Rifampicin–polymyxin B did not show synergistic activity against any subpopulation. On the other hand, triple combination with meropenem–rifampicin–polymyxin B presented synergistic effect against all subpopulations (Table 2). 

#### 2.2.3. *Galleria mellonella* Infection Model: Differences in Response to Antimicrobial Treatment between 798-1S and 798-4S Subpopulations

**Figure 4:**
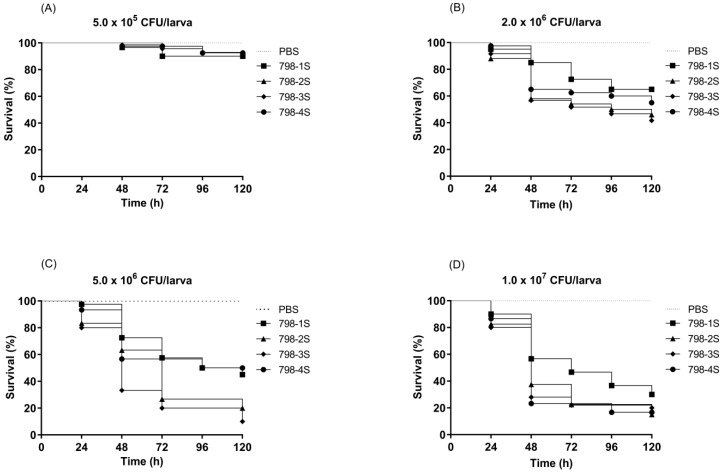
Survival curves of *G. mellonella* larvae inoculated with different inocula of *E. hormaechei* subsp. *oharae* (subpopulations 798-1S, 798-2S, 798-3S, and 798-4S): (**A**) 5.0 × 10^5^, (**B**) 2.0 × 10^6^, (**C**) 5.0 × 10^6^, and (**D**) 1.0 × 10^7^ CFU/larva.

In order to determine the *G. mellonella* susceptibility to *E. hormaechei* subsp. *oharae* infection, larvae were infected with four different bacterial inocula (5.0 × 10^5^, 2.0 × 10^6^, 5.0 × 10^6^ and 1.0 × 10^7^ CFU/larva) of subpopulations 798-1S, 798-2S, 798-3S, and 798-4S. It was verified that increasing doses of bacteria resulted in reduced larval survival in a dose-dependent manner during 120 h of incubation (Figure 4). No macroscopic changes or deaths were observed in the uninfected groups. Based on these data, 1.0 × 10^7^ CFU/larva was selected as the optimal inoculum for the subsequent treatment experiments, because this inoculum promotes staggered killing of approximately 80% of larvae covering the whole experimental period (Figure 4D). All subpopulations were pathogenic to *G. mellonella*. Before treating infected larvae, we evaluated toxicity of meropenem, polymyxin B, and rifampicin and after five days of incubation, all larvae injected with these antimicrobial agents were alive (data not shown). 

In vivo antimicrobial treatments were evaluated against 798-1S and 798-4S subpopulations. These subpopulations were chosen because they presented the most distinct virulence pattern in *G. mellonella*. The 798-1S showed a tendency to be less virulent (30% of survival after 120 h of incubation) and the 798-4S the more virulent (16.7% of survival) when larvae were inoculated with 1.0 × 10^7^ CFU.

**Figure 5:**
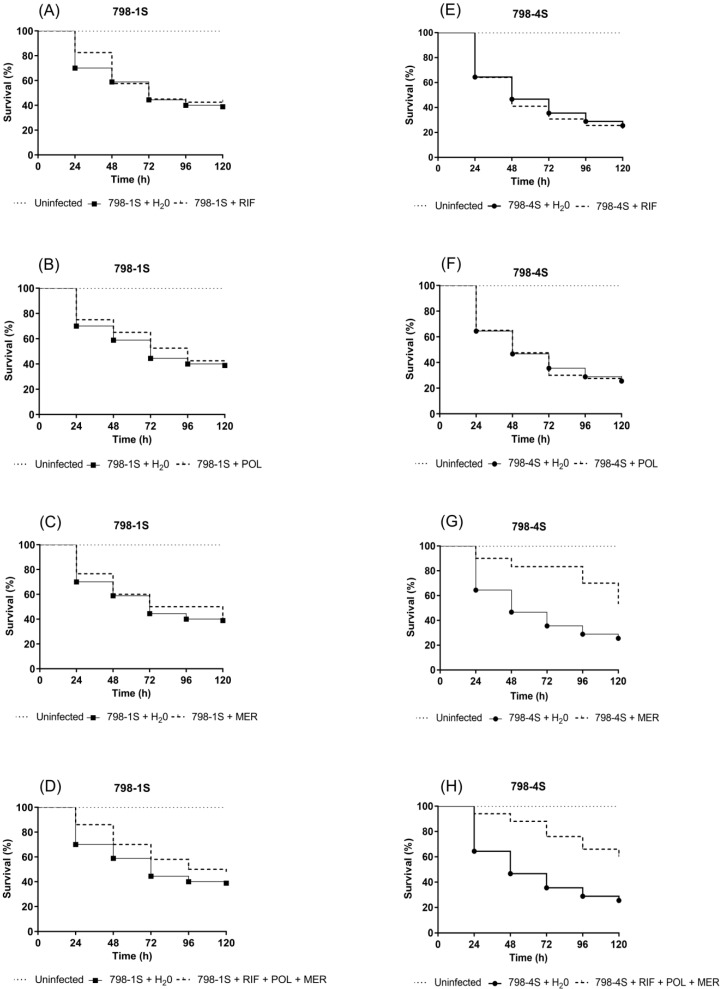
Survival curves for *G. mellonella* larvae inoculated with: (**A**–**D**) 798-1S and (**E**–**H**) 798-4S subpopulations following treatment with (**A** and **E**) RIF (20 mg/kg), (**B** and **F**) POL (3.0 mg/kg), (**C** and **G**) MER (85 mg/kg), or (**D** and **H**) RIF­–POL–MER combination. H_2_O: water; RIF: Rifampicin, POL: Polymyxin B, and MER: Meropenem.

Efficacy of monotherapy and triple antimicrobial combination in larvae infected with NDM-1 producing *E. hormaechei* subsp. *oharae* are shown in Figure 5. Meropenem alone (Figure 5G) or combined with polymyxin B plus rifampicin (Figure 5H) showed a significantly protective effect against 798-4S (*p* = 0.0010 and *p* < 0.0001, respectively). Conversely, none of the tested treatments was able to enhance the larvae survival when they were infected with 798-1S (Figure 5A–D), although this subpopulation has killed fewer larvae than the 798-4S.

## 3. Discussion

*Enterobacter hormaechei* subsp. *oharae* biofilm macrocolonies were able to generate genetically and phenotypically distinct subpopulations (Figure 1) that presented differences in virulence mechanisms and antimicrobial response. 

The amount of biofilm produced by subpopulations generated from the same macrocolony was different in five of the nine clinical isolates (Figure 2). Interestingly, the strongest biofilm producer isolate was obtained from urine (798). It is possible that this strain has additional virulence mechanisms that allow bacteria to adhere to bladder cells and cause urinary tract infection. Important virulence factors that contribute to biofilm formation are extracellular appendages called fimbriae. Genes encoding curli, type 1 fimbriae, and P pili were detected in all subpopulations of the group 798; however, the type 3 fimbriae gene was only detected in subpopulations able to produce biofilm (798-1S and 798-2S). On the other hand, subpopulations without *mrk*B (798-3S and 798-4S) did not produce biofilm in vitro using four different mediums (Figure 3A). As far as we know, there is no data showing the role of type 3 fimbriae in biofilm formation of *Enterobacter* spp.

Type 3 fimbriae are encoded by the *mrkABCDF* gene cluster [32] and were initially identified in *Klebsiella pneumoniae* strains by Duguid [33]. Since then, type 3 fimbriae have been described in other members of the Enterobacteriaceae family, including *Serratia* spp., *Enterobacter* spp., and *Escherichia coli* isolates [34,35,36]. In *K. pneumoniae*, this gene cluster is chromosomally encoded [37], while in other species it is found to be encoded by conjugative plasmids [36,38]. Type 3 fimbriae are involved in attachment to abiotic and biotic surfaces and in biofilm formation [39,40]. There is little information about type 3 fimbriae related with *Enterobacter* spp. in the literature. Adegbola and Old investigated type 1 and type 3 fimbriae in *Enterobacter* spp. and showed that type 1 fimbriae is most frequent and that most strains produced only one of these fimbriae [34]. Similar findings were described by Hornick et al., where most of *E. cloacae* respiratory isolates produced type 1 fimbriae, and fewer numbers also expressed type 3 fimbriae [35]. Recently, Azevedo et al. investigated nine virulence genes, including mrkD (adhesin type 3 fimbriae) and fimH (adhesive subunit of type 1 fimbriae), in eight *E. cloacae* isolates; surprisingly, no isolates presented virulence genes [41].

Macrocolony is well described and accepted as a biofilm model. Usually, studies evaluate macrocolonies’ structure and morphology, like the presence of wrinkle and ring patterns that are related with cellulose and curli fimbriae production, respectively [28,29,42,43,44,45,46]. As far as we know, Richer et al. (2014) were the first to investigated different subpopulations/regions generated by macrocolony [47]. In a macrocolony biofilm, bacteria typically conjugate with their closest neighbors when physical contact occurs between a donor and recipient cell to transmit horizontal plasmid, creating subpopulations that are independent from each other [30]. In this sense, some studies observed that a plasmid-bearing population can originate clonal sectors of plasmid-free cells [48,49]. This can explain why plasmid encoding *bla*_NDM-1_ did not spread throughout all the macrocolony and some subpopulations restored meropenem susceptibility. We could also hypothesize that the same occurred with type 3 fimbriae, since these fimbriae are encoded by plasmids in most Enterobacteriaceae species. 

Capability of bacteria to form biofilm on medical devices, such as catheter and prosthesis, has been proposed as one of the important mechanisms in nosocomially acquired and persistent infections, increasing resistance to antimicrobial treatment [16]. There are few studies evaluating biofilm formation in multidrug resistant strains. Recently, a study from Brazil demonstrated that the majority of repeated KPC infections are caused by the same strain that caused the previous infection/colonization, these findings illustrate the capacity of multiple clones producing biofilm to coexist in the same patient at the same time, serving as a constant reservoir of KPC in the hospital environment [50].

Regarding susceptibility profile, as expected, most subpopulations presented high antimicrobial resistance (Table 1). In vitro triple combination with meropenem–rifampicin–polymyxin B presented a synergistic effect against all tested subpopulations (Table 2). Corroborating with our findings, Tangden et al. tested 14 antimicrobial combinations using time-kill experiments against two NDM-1-producing *K. pneumoniae* strains, and found that the combination of rifampicin–meropenem–colistin was the most effective regimen [51]. In another study, Urban et al. showed that combination of polymyxin B–doripenem–rifampicin achieved 100% bactericidal activity for *Pseudomonas aeruginosa* and *E. coli*, 80% for K. pneumoniae, and 60% for *Acinetobacter baumannii* despite resistance to the carbapenems and rifampicin alone [52]. Although rifampicin by itself is not considered for the treatment infections caused by gram-negatives due to the rapid emergence of resistance, in vitro studies suggest that rifampicin has a synergistic activity when used as part of a combination therapy regimen against CPE [51,52,53,54,55]. 

In vivo, meropenem alone or combined with polymyxin B and rifampicin showed a significantly protective effect in larvae infected with 798-4S (*p* = 0.0010 and *p* < 0.0001, respectively). Conversely, none of the tested treatments was able to enhance the larvae survival when they were infected with 798-1S (Figure 5), although this subpopulation has shown a tendency to be less virulent then the 798-4S. Our hypothesis is that other bacterial factors, than biofilm, are being expressed in vivo and that the larval immune system can respond differently according to distinct antigenic stimuli evoked by bacterial subpopulations, influencing host survival.

Taken together, some discrepancies between in vitro and in vivo results were observed: (i) Polymyxin B was effective in vitro but not in vivo against 798-1S and 798-4S; (ii) triple combination presented synergistic effect in vitro but did not show significant enhancement in survival rates of larvae infected with 798-1S; and (iii) monotherapy with meropenem was able to increase the survival of larvae infected with 798-4S when compared with the control group treatment with water (53.3% × 25.6% survival), while in vitro evaluation classified 798-4S as resistant to this antimicrobial.

Supporting these results, discrepancies between in vitro and in vivo susceptibility for polymyxin B have been published in literature [56,57]. Yang et al., reported contradictory results considering in vitro and in vivo (*G. mellonella* model) colistin (polymyxin E) susceptibility in *A. baumanni* strains [56]. Moreover, Benthall et al. showed that the treatment with colistina in *G. mellonella* presented variable activity against *K. pneumoniae*, regardless of intrinsic susceptibility. In this same study, the carbapenems appeared to act better in vivo than in vitro, with meropenem able to clear infections caused by strains possessing *bla*_NDM-1_ and *bla*_VIM_ carbapenemases [58], similarly as observed herein for subpopulation 798-4S.

In summary, our findings demonstrate discrepancies between in vitro and in vivo susceptibility of *E. hormaechei* subsp. *oharae*. subpopulations to antimicrobial agents; some treatments were effective in vitro but not in vivo and vice versa. Interestingly, these subpopulations also showed different response to antimicrobial agents in *G. mellonella* infection model. Additionally, we may hypothesize that type 3 fimbriae are encoded on the plasmid and have a very important role in biofilm formation of *E. hormaechei* subsp. *oharae*. As far as we know, this is the first study evaluating macrocolonies of *E. cloacae* complex strains highlighting the potential of the macrocolony model as a tool to study the physiological heterogeneity within the biofilm. 

The results point out the ability of a multidrug resistant *E. hormaechei* subsp. *oharae* isolate in generating subpopulations, with distinct phenotypic and genetic features related to biofilm formation and antimicrobial profile. These findings might be associated to long term and chronic infections leading to an additional challenge in the treatment of bacterial infections and highlight the urgent need for newer antimicrobial development against biofilm related infection.

## 4. Materials and Methods

### 4.1. Bacterial Strains and Growth of Macrocolonies

Nine *E. hormaechei* subsp. *oharae* clinical isolates (Table 3) harboring *bla*_NDM-1_ gene (New Delhi metallo-beta-lactamase) were recovered from three hospitals and stored at −80 °C at the Laboratório de Pesquisa em Resistência Bacteriana of Hospital de Clínicas de Porto Alegre in Rio Grande do Sul, the southermost state of Brazil. These isolates were previously evaluated by Rozales et al. [59]. 

At first, clinical isolates were streaked on LB agar and incubated at 37 °C overnight. After checking purity of cultures, one colony was selected and dissolved in 5 mL of LB broth and incubated at same conditions. For macrocolonies’ growth, a volume of 3 uL of these cultures was spotted on LB agar plates supplemented with Congo Red (40 mg/L) and Coomassie Brilliant Blue (20 mg/L). The plates were incubated at 35 °C for 5 days [42,47].

After incubation, macrocolonies presented different areas, termed subpopulations. Cells were directly taken from these subpopulations and frozen in 10% skim milk with glycerol for further experiments. Before each experiment, these subpopulations were streaked on LB agar and incubated at 37 °C overnight.

### 4.2. Biofilm Formation: Microtiter Plates Assay

Biofilm formation was quantified using crystal violet (CV) assay in 96-well microtiter plates [60]. After 24 h of incubation, plates were washed to remove unbound bacteria and the attached bacteria were heat-fixed at 60 °C for 1 h. The biofilm was then stained with crystal violet, quantified by dissolving CV in 96% ethanol, and the optical density was measured at 595 nm (OD595). Biofilm formation was tested using different media: TSB, LB, LB supplemented with glucose (0.02 M), and M9 minimal medium supplemented with glucose (0.02 M) and MgSO_4_ (0.002 M). 

### 4.3. Polymerase Chain Reaction (PCR):Fimbrial Genes Detection

DNA of bacterial cells was extracted using boiling method, which is based on thermal shock and lysis of components other than nucleic acids. The same PCR cycling conditions used were for all genes: initial denaturation at 95 °C for 5 min followed by 30 cycles of denaturation at 94 °C for 30 s, annealing at 58 °C for 30 s, extension at 72 °C for 1 min, and a final extension at 72 °C for 5 min. Primer sets were design for PCR detection of fimbrial genes (Table 4).

### 4.4. Minimum Inhibitory Concentration (MIC): Agar Dilution Method

MIC of antimicrobial agents ceftazidim, ciprofloxacin, gentamicin, and meropenem was determined for all subpopulations using the agar dilution method according to EUCAST guideline [31]. The MIC was defined as the lowest concentration of the drug that inhibited growth of the tested bacteria. All MIC experiments were performed at least three times.

### 4.5. Checkerboard Assay

Minimum inhibitory concentration of meropenem, rifampicin, and polymyxin B and synergy with double and triple combinations were performed using the checkerboard method in 96-well microtiter plates with Mueller–Hinton broth and bacterial suspension containing approximately 5 × 10^5^ CFU/mL [61]. MIC and synergistic effect were visually determined as the lowest drug concentration (alone or combined, respectively) that inhibited bacterial growth. Susceptibility of meropenem and polymyxin B was interpreted according to EUCAST [31]. There is no standardized breakpoint value for rifampicin against Enterobacteriaceae according to guidelines. The synergism was determined by the fractional inhibitory concentration index (FICI) and interpreted as follows: FICI ≤ 0.5 indicates synergy; FICI > 0.5 ≤4 = no interaction; FICI > 4.0 = antagonism [61,62].

### 4.6. Galleria mellonella Model Studies

The whole cycle of *G. mellonella* was maintained in our laboratory at 28 °C. Insects were fed with an artificial diet consisting of honey and several flours. Larvae weighting 220–260 mg were randomly selected to comprise groups of ten larvae which were inoculated with 10 uL of bacterial suspension by injection into the haemocoel via the last right proleg, using a Hamilton syringe (Sigma-Aldrich). Four different bacterial suspensions in sterile phosphate-buffered saline (PBS) were tested for each subpopulation: 5.0 × 10^5^, 2.0 × 10^6^, 5.0 × 10^6^ and 1.0 × 10^7^ CFU/larva. Uninfected larvae (either uninoculated or injected with only PBS) were used as negative controls. Afterwards caterpillars were incubated in Petri dishes at 37 °C and were observed daily during 120 h. They were considered dead when they did not respond to touch. 

Based on larvae survival curve with different inocula, bacterial concentration of 1.0 × 10^7^ CFU/larva was selected to be used for evaluating treatment efficacy. Antimicrobial agents (10 uL) were administered as single injection into the last left proleg 30 min after bacterial inoculation. Antimicrobial doses were selected to be representative of those used to treat human infection: polymyxin B at 3.0 mg/kg, meropenem at 85 mg/kg, and rifampicin at 20 mg/kg [13].

### 4.7. Statistical Analysis

Larvae survival data were plotted using the Kaplan–Meier curve and comparisons between groups were made using the log-rank test (Graphpad Prism 6 software). Biofilm formation of subpopulations generated from the same macrocolony was compared using two-way ANOVA. All analyses were performed on at least three independent experiments using Graphpad Prism 6 software. In all tests, *p* ≤ 0.05 was considered significant.

## Figures and Tables

**Table 1:** Minimum inhibitory concentration of antimicrobial agents against *E. hormaechei* subsp. *oharae* subpopulations.

Subpopulations	CAZ	CIP	GEN	MER
7-1 S	>256	8	≤2	32
7-2 S	>256	8	≤2	64
7-3 S	32	8	≤2	≤2
7-4 S	>256	8	≤2	32
67-1 S	>256	>256	256	64
67-2 S	>256	>256	256	256
104-1 S	32	128	32	≤2
104-2 S	32	128	16	≤2
104-3 S	32	128	16	≤2
245-1 S	128	64	>256	≤2
245-2 S	64	64	>256	≤2
245-3 S	64	64	>256	≤2
798-1 S	>256	16	256	32
798-2 S	>256	32	>256	32
798-3 S	>256	32	256	16
798-4 S	>256	8	128	16
821-1 S	64	>256	>256	≤2
821-2 S	>256	256	>256	32
821-3 S	>256	128	32	64
821-4 S	>256	256	>256	32
977-1 S	256	8	256	≤2
977-2 S	>256	8	256	≤2
997-1 S	>256	8	≤2	≤2
997-2 S	>256	8	≤2	≤2
1105-1 S	>256	64	>256	32
1105-2 S	>256	64	256	64
1105-3 S	128	64	256	≤2

Highlighted rows presented macrocolonies that lost the *bla*_NDM-1_ gene and became susceptible to meropenem. CAZ: Ceftazidim, CIP: Ciprofloxacin, GEN: Gentamicin, MER: Meropenem. Minimum inhibitory concentrations are in µg/mL.

**Table 2:** Minimum inhibitory concentration and checkerboard results of meropenem, polymyxin B, and rifampicin alone and combined against NDM-1 producing *Enterobacter hormaechei* subsp. *oharae* subpopulations.

Subpopulation	MIC (µg/mL)			Checkerboard (interpretation)			
	MER	POL	RIF *	MER/POL	MER/RIF	POL/RIF	MER/POL/RIF
798-1S	32 (R)	2 (S)	128	4/0.5 (SE)	2/8 (SE)	1/64 (NI)	2/0.25/4(SE)
798-2S	32 (R)	1 (S)	128	8/0.5 (NI)	0.5/64 (NI)	1/1 (NI)	1/0.25/4 (SE)
798-3S	16 (R)	1 (S)	128	4/0.5 (NI)	1/64 (NI)	0.5/64 (NI)	1/0.25/4 (SE)
798-4S	16 (R)	1 (S)	128	1/1 (NI)	2/64 (NI)	0.5/64 (NI)	1/0.25/4 (SE)

MER: Meropenem, POL: Polymyxin B, RIF: Rifampicin; R: Resistant; S: Susceptible; SE: Synergistic effect, NI: No interaction. Synergistic effects are highlighted in gray. * There is no breakpoint for rifampicin against Enterobacteriaceae according to EUCAST.

**Table 3:** Bacterial strains used in this study.

*E. hormaechei* subsp. *oharae* Strains	Description
**1** (245)	Sink isolate
**2** (7)	Rectal swab isolate
**3** (67)	Rectal swab isolate
**4** (104)	Rectal swab isolate
**5** (798)	Urine isolate
**6** (821)	Cerebrospinal fluid isolate
**9** (977)	Rectal swab isolate
**10** (997)	Rectal swab isolate
**11** (1105)	Rectal swab isolate

In bold: Strains identification according to Rozales et al. (2014).

**Table 4:** Primers used in this study.

Gene	Encoding Protein	Primer Sequence (5’ to 3’)	Size (bp)
*csg*A	Major fimbrial subunit	Forward: caacctgatgcacagtcacc	214
		Reverse: tggacagggatctgatgaca	
*csg*B	Minor subunit	Forward: agccatttgcgactgtctct	233
		Reverse: tgtccgttatttcccaggag	
*csg*D	Transcriptional regulator of the *csgBAC* operon	Forward: ccttccttacaagcgacagc	236
		Reverse: tcgcggaaaggatactcatc	
*fim*A	Major fimbrial subunit	Forward: tgctgtcgaggatctcaatg	229
		Reverse: acggttaatctcggccagta	
*fim*H	Fimbrial adhesion	Forward: ccccgtccagatagtcgtta	210
		Reverse: acgacctgacggacaaattc	
*pap*C	Fimbrial usher	Forward: ccctgaagaccgatgacaat	148
		Reverse: cggaacggaggtttgataga	
*pap*D	Fimbrial chaperone	Forward: tggatggaagacgagaaagg	134
		Reverse: catccagtacagcgtctcg	
*mrk*B	Fimbrial chaperone	Forward: ggtggctgaatctgctggaaatt	514
		Reverse: atcacggttttactgttcagggcttt	
		Reverse: attggcataagtcgcaatcc	

*csg*A, *csg*B, and *csg*D: Curli genes; *fim*A and *fim*H: Type 1 fimbriae genes; *pap*C and *pap*D: P pili genes; *mrk*B: Type 3 fimbriae gene.
